# St. Louis Dental Society

**Published:** 1888-12

**Authors:** Wm. Conrad

**Affiliations:** Corresponding Secretary. 321 N. Grand Avenue.


### ST. LOUIS DENTAL SOCIETY.

ST. LOUIS, MO., January 5th, 1889.

The Annual Meeting of this society was held at the office of Dr. A. J. Prosser, 3109 Olive Street, Wednesday Evening, January 2d, and the following officers were elected for 1889:

*President*—Dr. A. J. Prosser.

*Vice-President*—Dr. J. Warren Wick.

*Recording Secretary*—Dr. Jesse E. Grosheider.

*Corresponding Secretary*—William Conrad.

*Treasurer*—Dr Henry Fisher.

*Publication Committee*—Drs. H. H. Keith, John G. Harper, and J. B. Vernon.

*Committee on Ethics and Elections*—Drs. J. B. Newby, Wm. N. Morrison, and A. H. Fuller.

There were seventeen meetings held, and fourteen papers presented during 1888.

WM. CONRAD,

321 N. Grand Avenue. *Corresponding Secretary.*

